# Structure Identification of *Ganoderma lucidum* Spore Polysaccharides and Their Antitumor Activity In Vivo

**DOI:** 10.3390/molecules29102348

**Published:** 2024-05-16

**Authors:** Hui-Min Liu, Jun Cheng, Xiao-Yi Wang, Yan Jiang, Jia Ni, Yun Zhang, Wei Wang

**Affiliations:** 1 School of Perfume & Aroma and Cosmetics, Shanghai Institute of Technology, Shanghai 201418, China; 2 Engineering Research Center of Perfume & Aroma and Cosmetics, Ministry of Education, Shanghai 201418, China; 3 Shanghai Institute of Organic Chemistry, Chinese Academy of Sciences, 345 Lingling Road, Xuhui District, Shanghai 200032, China

**Keywords:** efficacy, structural nature, *Ganoderma lucidum* spore polysaccharide, antitumor activity

## Abstract

*Ganoderma lucidum* spore powder, valued for its nutritional and medicinal properties, contains polysaccharides crucial for its efficacy. However, the complex structural nature of these polysaccharides necessitates further investigation to fully realize their potential. This study aimed to investigate the effects of acid heat treatment on *Ganoderma lucidum* spore polysaccharides (GLSPs) to enhance their properties and application in antitumor activity. The GLSP was obtained via acid heat treatment, concentration, and centrifugal separation. This process led to a notable reduction in polysaccharide molecular weight, increasing water solubility and bioavailability. Analytical techniques including NMR spectroscopy and methylation analysis revealed a polysaccharide composition comprising four distinct monosaccharides, with molecular weights of 3291 Da (Mw) and 3216 Da (Mn). Six different linkage modes were identified, with a molar ratio of 1:5:2:3:4:3. In vivo experiments demonstrated the GLSP’s significant inhibitory effect on the growth of four tumor models (sarcoma S180, Lewis lung cancer, liver cancer H22, and colon cancer C26) in mice, with no observed toxicity. These findings suggest the GLSP’s potential as an antitumor therapeutic agent for clinical use.

## 1. Introduction

As one of the leading causes of disease in the world today, cancer remains a major global public health problem [1]. In 2018, there were 10.18 million new cancer cases and 9.6 million cancer deaths worldwide [2]. This includes early-onset cancers that are not limited to patients over the age of 50, and a younger age at the onset of cancer is increasingly becoming an epidemic trend [3]. At present, the treatment of cancer mainly includes radiotherapy, chemotherapy, surgery, and so on [4]. As a complementary and alternative medicine in Western countries and a traditional medical method in Asia, Chinese medicine is attracting global attention in the field of life sciences [5]. In addition to protein and nucleic acid, polysaccharides are another kind of biological macromolecule that play an important role in the body, and they are also an indispensable part of the body [6]. Polysaccharides are widely distributed in nature and are closely related to many pathologic processes and aging processes [7]. It has been found that polysaccharides have a significant effect on the treatment of tumors and have become an important research hotspot in the development of high-efficiency and low-toxicity antitumor drugs in recent years [8].

*Ganoderma lucidum*, as a valuable medicinal fungus, is also one of the “medicine and food homology” health herbs in China, with a long history of medicinal use and high nutritional value [9]. The literature has confirmed that *Ganoderma lucidum* has a wide range of pharmacological effects, such as antitumor, immune regulation [10], anti-inflammatory, antioxidant, and anti-aging effects [11], and has an improving effect on a variety of diseases such as the nervous system [12], cardiovascular system, and digestive system [13]. Due to the huge pharmacological potential of *Ganoderma lucidum* spore polysaccharides (GLSPs) and the development of groundbreaking technology, the antitumor effect of GLSPs has received extensive attention. Song M et al. revealed that GLSPs promoted cell polarization into the M1 type, increased the expression level of cytokines (TNF-α, IL-1β, IL-6, and TGF-β1), and blocked tumor cells in G2/M phase by activating macrophages. It also activated the PI3K/AKT signaling pathway, affected the mitochondrial apoptosis pathway, and promoted the apoptosis of tumor cells [14]. A water-soluble polysaccharide was isolated from a *Ganoderma lucidum* spore by Fu ZL et al. with the average molecular weight of the polysaccharide being 1.5 × 10^4^ Da, showing a significant inhibition of S180 tumor growth in vivo [15]. Li D et al. found the effect of GLSPs on the recovery of small intestinal barrier function in the paclitaxel (PTX)-induced mouse breast cancer model and IEC-6 cell line [16]. Zhong JY et al. demonstrated GLSP-induced apoptosis in AGS cells, implicating the regulation of Bcl-2, caspase-3, and lytic PARP expression, as well as the modulation of autophagy flux, suggesting potential for gastric cancer treatment [17]. Moreover, Wang PY et al. suggested that GLSPs’ antitumor activity is linked to immune response stimulation involving NK cells, T cells, and macrophages [18]. Shi YJ et al. corroborated GLSPs’ inhibitory effect on various cancers in vitro and validated their antitumor efficacy using zebrafish models [19]. However, despite these advancements, a comprehensive understanding of GLSPs’ structure–function relationship remains elusive, particularly regarding polysaccharides with molecular weights below 10,000 Da.

Our study aims to explore the impact of acid heat treatment on *Ganoderma lucidum* polysaccharides, with a specific focus on elucidating the structural modifications and their implications for antitumor activity. We hypothesize that acid heat treatment will facilitate the release and dissolution of polysaccharides from *Ganoderma lucidum* spores, resulting in lower-molecular-weight polysaccharides (<5000 Da) with enhanced bioavailability [20]. Subsequently, we anticipate observing potent antitumor effects of GLSPs in various murine tumor models, providing valuable insights into their therapeutic potential and paving the way for the development of novel antitumor drugs or health products with improved efficacy and safety profiles.

Through our investigation, we aim to contribute to the growing body of knowledge surrounding the structural characteristics and therapeutic applications of *Ganoderma lucidum* polysaccharides, ultimately advancing the field of cancer therapeutics and offering new avenues for clinical intervention. 

## 2. Materials and Methods

### 2.1. Materials and Reagents

The *Ganoderma lucidum* spore powder utilized in this study was procured from the Huangshan District Ganoderma planting base. Kunming mice and C57BL/6 mice were purchased and provided by Shanghai Slack Laboratory Animal Liability Co., Ltd., (Shanghai, China), weighing 18–22 g, both male and female, and the same sex was used in each batch of experiments. There were 6 naked mice in each group of the experimental group and positive control group, 8–10 mice in each group of other mice, and 2 groups in each negative control group. Mouse sarcoma S180, mouse Lewis lung cancer, mouse liver cancer H22 and mouse colon cancer C26, cell or in vivo tumors were all maintained by the pharmacological Department of Shanghai Institute of Pharmaceutical Industry. Cyclophosphamide for injection (CTX), produced by Jiangsu Hengrui Pharmaceutical Co., Ltd., (Lianyungang, China), batch No. 07040621; 5-fluorouracil (5-Fu), produced by Shanghai Xudong Haipu Pharmaceutical Factory, batch No. 070307; Cisplatin (DDP), Jinzhou Jiutai Pharmaceutical Co., Ltd., (Jinzhou, China), lot number 060404. All other chemicals and reagents used were analytical grade.

### 2.2. Extraction and Purification of GLSP

In view of the difficulty in extracting polysaccharides from *Ganoderma lucidum* spores, organic or inorganic acid was used to heat sporangium powder for the first time in this paper [21]. As the acid penetrated the double walls of *Ganoderma lucidum* spore powder under heating conditions, many micropores were formed on the spore wall, and the acid solution entered the micropores to degrade the macromolecular polysaccharides inside the sporangium powder. After degradation, the molecular weight of the polysaccharide decreased, and the water solubility increased. Water-soluble polysaccharides can be released through micropores, then extracted and purified by water extraction and alcohol precipitation.

*Ganoderma lucidum* spore powder underwent treatment by immersion in aqueous solutions of inorganic acids ranging from 0.1 to 2 mol/L concentration. The mass-to-volume ratio of *Ganoderma lucidum* spore powder to inorganic acid solution varied between 100 and 800 g/L, while the stirring was maintained at temperatures of 60 to 100 °C for durations spanning from 10 min to 4 h. Following the reaction, inorganic alkali compounds such as phosphoric acid, sulfuric acid, hydrochloric acid, or nitric acid were introduced to neutralize the spore powder to a pH range of 5 to 7.5. Alternatively, *Ganoderma lucidum* spore powder could be subjected to treatment with organic acids or their aqueous solutions, ranging from 50 to 100%. The mass-to-volume ratio of *Ganoderma lucidum* spore powder to organic acid or organic acid solution was maintained between 100 and 800 g/L, with the reaction occurring at temperatures ranging from 60 °C to reflux for durations of 10 min to 4 h. The resultant acid-treated spore powder was obtained via solvent evaporation under reduced pressure. Subsequently, the acid-treated sporangium powder was immersed in water for soaking purposes, with durations ranging from 2 to 24 h and a mass ratio of 1:6 to 15 of *Ganoderma lucidum* spore powder to water. The soaking solution could be concentrated as desired, and precipitation was induced by adding 3 to 8 times the volume of an organic solvent such as methanol, ethanol, or acetone. The resulting precipitate was then dissolved in water.

The subsequent extraction procedure of GLSP employed in this study involved the following steps: Initially, 50 g of *Ganoderma lucidum* spore powder was dispersed into a 500 mL solution of 0.1 mol/L sulfuric acid. The resulting mixture was then subjected to heating at 100 °C with continuous stirring for a duration of 4 h. Subsequent to the reaction, neutralization was achieved by the addition of barium hydroxide until the pH reached 7.5, yielding acid-treated *Ganoderma lucidum* spore powder. This material was further treated by soaking it in 500 mL of water for a period of 2 h, followed by the filtration and concentration of the soaking solution to 50 mL. Ethyl alcohol (150 mL) was subsequently added to precipitate the polysaccharide, which was then dissolved in water. The resulting solution was subjected to centrifugation to obtain a supernatant, which was further concentrated and dried to yield the final product. 

### 2.3. Structure Analysis of GLSP

#### 2.3.1. Fourier Transform-Infrared (FT-IR) Analysis

According to the method proposed by Bolade et al. [22], a Fourier infrared spectrometer was used to measure the transmittance [23], and 2 mg GLSP was mixed with dry KBr powder and pressed into tablets, and the FT-IR spectrum of the GLSP was obtained by scanning with a PerkinElmer FT-IR spectrometer in the range of 4000–500 cm^−1^.

#### 2.3.2. Molecular Weight Determination

Based on the Shimadzu LC-10A system, equipped with a BRT105–103-101 tandem gel column (8 mm × 300 mm) and RI-20A differential detector, the homogeneity and molecular weight of the GLSP were detected by HPGPC [24]. For each run, 25 µL 5 mg/mL sample solution was first injected, and then the sample was eluted using a mobile phase (0.05 mol/L NaCl) at a flow rate of 0.8 mL/min and a temperature of 40 °C. The chromatogram and retention time (RT) of the GLSP were obtained, and the lgMw-RT and lgMn-RT calibration curves were drawn using the known maltoheptaose with weight average molecular weight (Mw) and number average molecular weight (Mn) and different commercially available glucan standards. The Mw, Mn, and polydispersity index (PDI = Mw/Mn) were obtained by calculating the retention time [25].

#### 2.3.3. Monosaccharide Composition Analysis

The monosaccharide composition was determined by ion chromatography [26]. A total of 5 mg of the GLSP was hydrolyzed with 3 mol/L trifluoroacetic acid (TFA) in a sealed tube at 120 °C for 3 h, and the acid hydrolysis solution was accurately absorbed and transferred to the tube for nitrogen drying. In order to remove the residual TFA, 5 mL of distilled water was added to the hydrolysate after hydrolysis and mixed well, and 20 µL was absorbed, and 980 µL of deionized water was added. Then, it was centrifuged at 12,000 rpm for 5 min, and then the CarbopacTMPA20 high performance anion exchange column (3 mm × 150 mm, 25 µL) was used. The supernatant of the hydrolyzed GLSP was analyzed by the ICS 5000 ion chromatography system of Dionex and then detected by an electrochemical detector. The samples were eluted with distilled water (mobile phase A), 15 mmol/L NaOH (mobile phase B), 15 mmol/L NaOH, and 100 mmol/L NaAc (mobile phase C) at a flow rate of 0.3 mL/min and at 30 °C. For the quantitative determination of monosaccharides, the monosaccharide standard was prepared according to the corresponding response factor and total peak area. Sixteen monosaccharide standards (fucose, rhamnose, arabinose, galactose, glucose, xylose, mannose, fructose, ribose, galacturonic acid, glucuronic acid, galactosamine hydrochloride, glucosamine hydrochloride, *n*-acetyl-d glucosamine, guluronic acid, mannose acid) were prepared into standard mother solutions. The concentration standard of each monosaccharide standard solution was selected as the mixed standard. According to the absolute quantitative method, the mass of different monosaccharides was determined, and the molar ratio was calculated according to the molar mass of monosaccharides.

#### 2.3.4. Methylation Analysis

The partially methylated acetyl derivatives of sugar alcohols were obtained and analyzed by combined gas chromatogram–mass spectrometry (GC-MS) to determine the linking mode of each monosaccharide residue [27]. A total of 2 mg of the dried GLSP was dissolved in 500 µL of DMSO, 1 mg of NaOH was added and incubated for 30 min, 50 µL of CH_3_I solution was added for 60 min, and then the free hydroxyl of polysaccharides was methylated. After the methylation, the polysaccharides were hydrolyzed to monosaccharides with 2 mol/L TFA at 121 °C for 90 min, and then the monosaccharides were reduced to polyols with NaBD_4_, and the remaining hydroxyl groups of the methylated polyols were acetylated and dissolved in chloroform with acetic anhydride, which was analyzed by GC-MS. The analytical instrument used in this experiment was the 7890A-5977B G4567A (Agilent Technologies Inc., Santa Clara, CA, USA) G4567A automatic sampler. The chromatographic system was the Agilent gas chromatography system (Agilent 7890A; Agilent Technologies, Santa Clara, CA, USA), Agilent BPX70 column (30 m × 0.25 mm × 0.25 µm, SGE, Lingwood, Australia). The sample size was 1 µL, the shunt ratio was 10:1, the carrier gas was high-purity helium, and the carrier gas flow rate was 1.5 mL/min. The initial temperature of the column temperature chamber was 140 °C for 2 min, and the temperature was heated to 230 °C at 3 °C/min for 3 min. Agilent Company’s four-pole mass spectrometry system was used (Agilent 5977B; Agilent Technologies, Santa Clara, CA, USA) with an electron-bombarded ion source (EI). Using an electron bombardment ion source (EI), the analytes were detected in full SCAN mode with a mass scan range (*m*/*z*) of 30–600.

#### 2.3.5. NMR Spectroscopy Analysis

In order to further elucidate the structural characteristics of the polysaccharide samples, comprehensive nuclear magnetic resonance (NMR) spectroscopy analyses were conducted. The analyses included one-dimensional ^1^H NMR and ^13^C NMR, along with two-dimensional ^1^H–^1^H COSY Correlation Spectroscopy (COSY), Heteronuclear Single Quantum Coherence (HSQC), and Heteronuclear Multiple Bond Correlation (HMBC) techniques. The lyophilized samples of the polysaccharides were fully dissolved in 0.5 mL of deuterated water (D_2_O) to prepare for spectroscopic evaluation. These solutions were then analyzed using a Bruker AVANCE HD III nuclear magnetic resonance spectrometer operating at 600 MHz. This instrument is equipped to handle high-definition ^1^H NMR and ^13^C NMR analyses, ensuring precise and clear spectral data.

For statistical evaluation, we performed t-tests to assess the significance of any observed differences or trends in the NMR spectral data. The choice of t-tests was based on their suitability for comparing means between two groups, which aligns with our analytical objectives. All calculations were conducted using [insert name of statistical software], ensuring transparency and reproducibility in our statistical assessment.

### 2.4. Antitumor Effects of GLSPs on Multiple Tumor Models

Mice of the same sex were used in each experiment. *Ganoderma lucidum* polysaccharides for injection were added to quantitative normal saline to dissolve, and the appropriate amount of normal saline was gradually added after direct and precise measurement and diluted to the required concentration. Dosage volume 0.2 mL/20 g BW. Four tumor models were used, namely mouse sarcoma S180 (subcutaneous axillary inoculation model), mouse Lewis lung cancer (subcutaneous inoculation model of foot), mouse liver cancer H22 (subcutaneous axillary inoculation model), and mouse colon cancer C26 (subcutaneous axillary inoculation model). Each model was randomly divided into 5 groups (Numbers. every group = 8/10 mice): the negative control group was selected as normal saline, the positive control group was selected as cyclophosphamide (CTX), cisplatin (DDP), and 5-fluorouracil (5-Fu), and the different doses of GLSPs were selected as high, medium, and low doses (50, 25, and 12.5 mg/kg, respectively), iv × 14–15 qd regimen. All procedures were carried out in strict accordance with the laws and regulations on the use and care of laboratory animals and the guidelines formulated by the Shanghai Institute of Pharmacy and were approved by the Committee of the Institute of Animal Experiments.

The experiment involved an axillary subcutaneous inoculation model and toe subcutaneous inoculation model. Subcutaneous axillary inoculation model: The tumor source with strong growth was taken under aseptic conditions, and cell suspension was prepared by the homogenization method or cultured cells were used, and the concentration was about 1–2 × 10^7^/mL. The tumor cells were injected subcutaneously into the right axillary of Kunming mice, the volume of tumor cells was 0.2 mL/mouse, and the drug was administered according to the experimental design. The animals in each group were killed about three weeks ago, and the tumors were dissected and weighed, and the tumor inhibition rate was calculated. Foot subcutaneous inoculation model: Lewis lung cancer with vigorous growth was extracted under aseptic conditions, prepared by the homogenization method into about 2 × 10^7^/mL cell suspension, and 0.05 mL/mouse (about 1 × 10^6^ tumor cells) was inoculated subcutaneously on the right toe of C57BL/6 mice the next day, according to the experimental design; the animals in each group could be killed directly in about two weeks, and the toes bearing the tumor were removed and weighed. The tumor inhibition rate was calculated.

The antitumor effect of the subject was observed dynamically by measuring the diameter of the tumor. The number of times the tumor diameter was measured depends on the growth of the transplanted tumor, usually 2 to 3 times per week. According to the measured results, the relative tumor volume (RTV) was calculated as follows: RTV = V_t_/V_0_, where V_0_ is the tumor volume measured at the time of separate cage administration, and V_t_ is the tumor volume measured at each measurement. The evaluation index of antitumor activity was the relative tumor growth rate T/C (%), calculated as follows: T/C (%) = (T_RTV_/C_RTV_) × 100% (T_RTV_: RTV in treatment group; C_RTV_: negative control RTV). Evaluation of antitumor activity: T/C (%) > 60 is ineffective, ≤60 is effective (generally refers to the standard of chemotherapy drugs). The tumor growth inhibition value (TGI) = (1 − T/C) × 100%.

## 3. Results and Discussion

### 3.1. Molecular Weight of GLSPs

**Figure 1:**
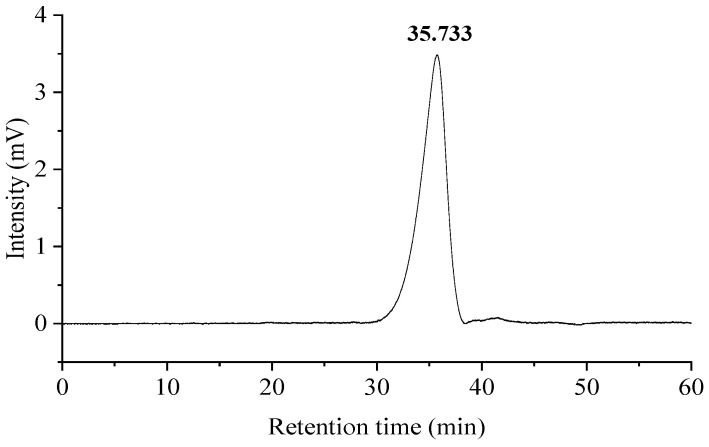
High-performance gel permeation chromatogram of GLSP.

According to the HPGPC chromatogram of the GLSP, there is a single symmetric peak (Figure 1), indicating that the GLSP has good homogeneity. The correction curve equations of Mw and Mn are y = −0.222x + 11.45 (R^2^ = 0.994) and y = −0.222x + 11.44 (R^2^ = 0.994), respectively. Based on the retention time (RT) of 35.733 min, the Mw and Mn of the GLSP are 3291 Da and 3216 Da, respectively, and the PDI value is 1.02.

### 3.2. FT-IR and Monosaccharide Composition Analysis of GLSPs

**Figure 2:**
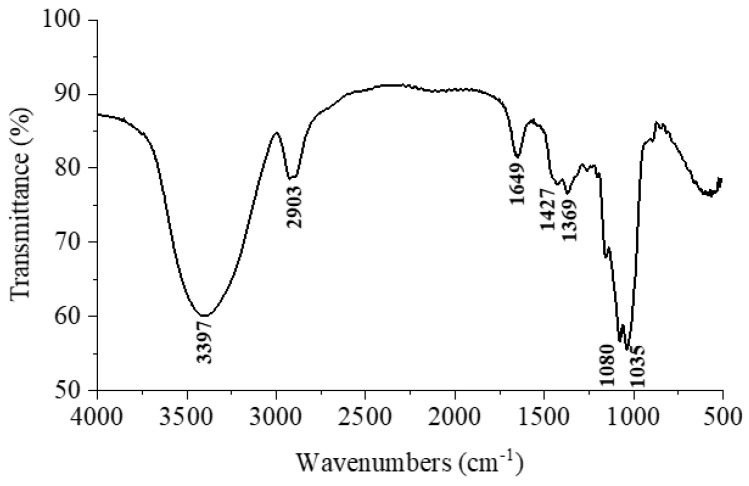
The FTIR spectrum of the GLSP.

The infrared spectrum (FT-IR) of the GLSP is shown in Figure 2, and the results show that the GLSP has characteristic absorbance of polysaccharide chemical bonds and functional groups. The wide characteristic absorption peak at wavenumber 3397 cm^−1^ is a common O-H bond peak in carbohydrate compounds, which is attributed to a large amount of O-H stretching vibration in the GLSP. The signal at 2903 cm^−1^ is the C-H bond peak in the sugar ring, which is caused by the stretching vibration of C-H in the GLSP. Peaks observed at wavenumber 1649 cm^−1^ correspond to the expansion vibration of the COO- bond. Peaks at 1427 cm^−1^ and 1369 cm^−1^ signify the bending vibration associated with the O-H bond. Additionally, the absorption peaks at wavenumbers 1080 cm^−1^ and 1035 cm^−1^ are indicative of a pyranose ring, attributable to the vibration of the C-O-C bond.

**Figure 3:**
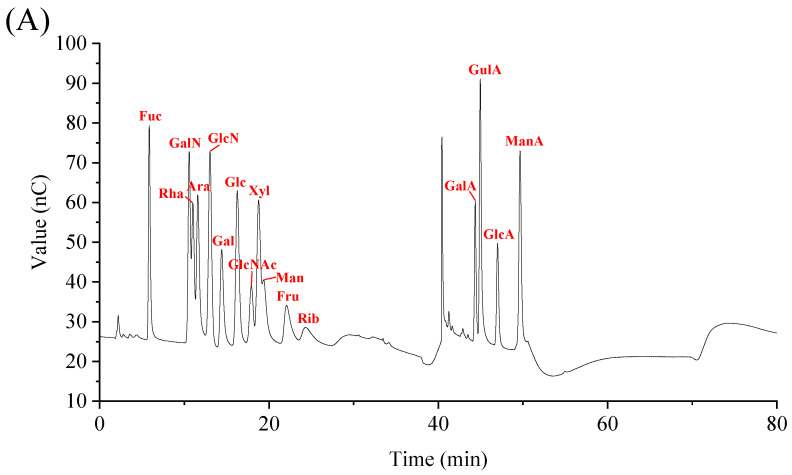

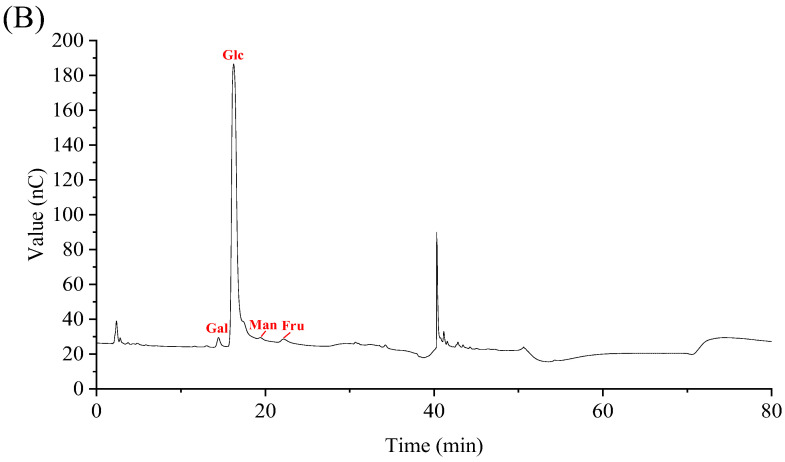
(**A**) Ion chromatogram of 16 monosaccharides of mixed standards. (**B**) Ion chromatogram of GLSPs.

The monosaccharide composition of the GLSP was determined using ion chromatography (Figure 3B), with a comparative analysis of its spectra against standard products (Figure 3A). The analysis revealed that the GLSP is composed of galactose, glucose, mannose, and fructose. Further examination from Table 1 indicates that glucose predominates, constituting 87.5% of the GLSP and suggesting its role as the primary structural component or “skeleton” of the GLSP. Minor quantities of fructose (8.7%), galactose (3.1%), and mannose (0.7%) were also detected, implying the heteropolysaccharide nature of the GLSP.

### 3.3. Methylation and NMR Analysis

In order to further identify the definitive structure of polysaccharides, an examination of the glucoside bonds was conducted using methylation coupled with GC-MS analysis. As shown in Table 2, according to the MS spectrum of GC-MS, the retention time of 12.655 min is Glcp–(1→, the retention time of 13.314 min is →3)–Glcp–(1→ and →4) –Glcp–(1→, the retention time of 13.529 min is →6)–Glcp–(1→, The retention time of 14.150 min is →3,6)–Glcp–(1→. Thus the glucan is mainly by the Glcp–(1→, →3)–Glcp–(1→, →4)–Glcp–(1→, →6)–Glcp–(1→ and →3,6)–Glcp–(1→ five kinds of connection mode, the mole ratio is about 3:4:5:3:3.

**Figure 4:**
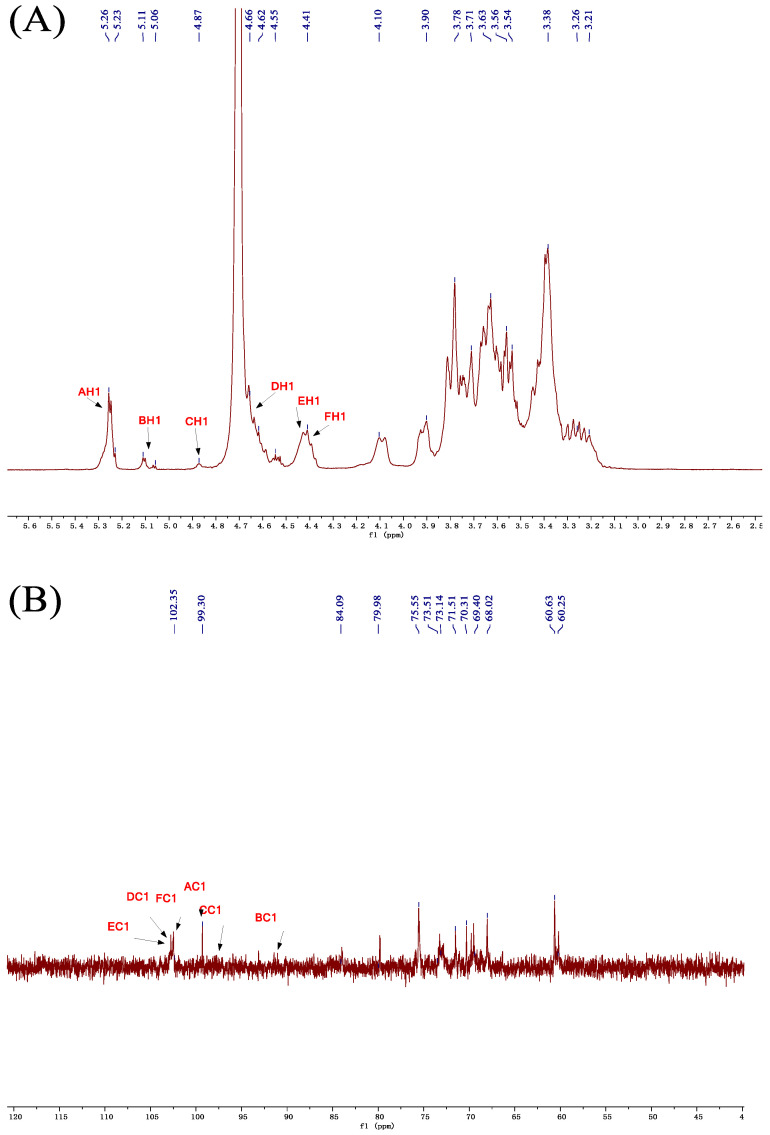
1D-NMR spectra of GLSP. (**A**) ^1^H spectrum; (**B**) ^13^C spectrum.

The structural and compositional details of the polysaccharide were confirmed using a combination of one-dimensional (1D) and two-dimensional (2D) NMR spectra, with visual representations provided in the figures [28]. In the ^1^H NMR spectrum (Figure 4A), within the anomeric region (4.3–5.5 ppm), multiple sets of anomeric proton signals were observed. The prominent peaks were located at 5.26, 5.23, 5.11, 5.06, 4.87, 4.66, 4.55, and 4.41 ppm, indicating the diversity of sugar residues resulting from methylation [29]. Outside this region, in the 3.0–4.2 ppm range, typical proton signals associated with non-anomeric protons of sugar rings were identified, displaying the complex nature typical of polysaccharide hydrogen spectra [30].

In the ^13^C NMR spectrum (Figure 4B), the absence of signals corresponding to uronic acid carbonyl carbon atoms in the range of 170–220 ppm provided evidence for the polysaccharide’s neutral pH [31]. Within the spectrum’s anomeric region (90–110 ppm), multiple sets of signals originating from anomeric carbon atoms were observed at 102.50, 99.26, 99.71, 95.40, and 91.48 ppm. These signals were indicative of the presence of diverse sugar residues. Additionally, carbon signals within the range of 55–85 ppm were identified, with signals at 58–65 ppm attributed to the C-6 methylene-CH_2_-carbon of pyranose-type sugar residues. This observation underscores the structural complexity of the polysaccharide, characterized by the predominance of glucopyranose units. 

**Figure 5:**
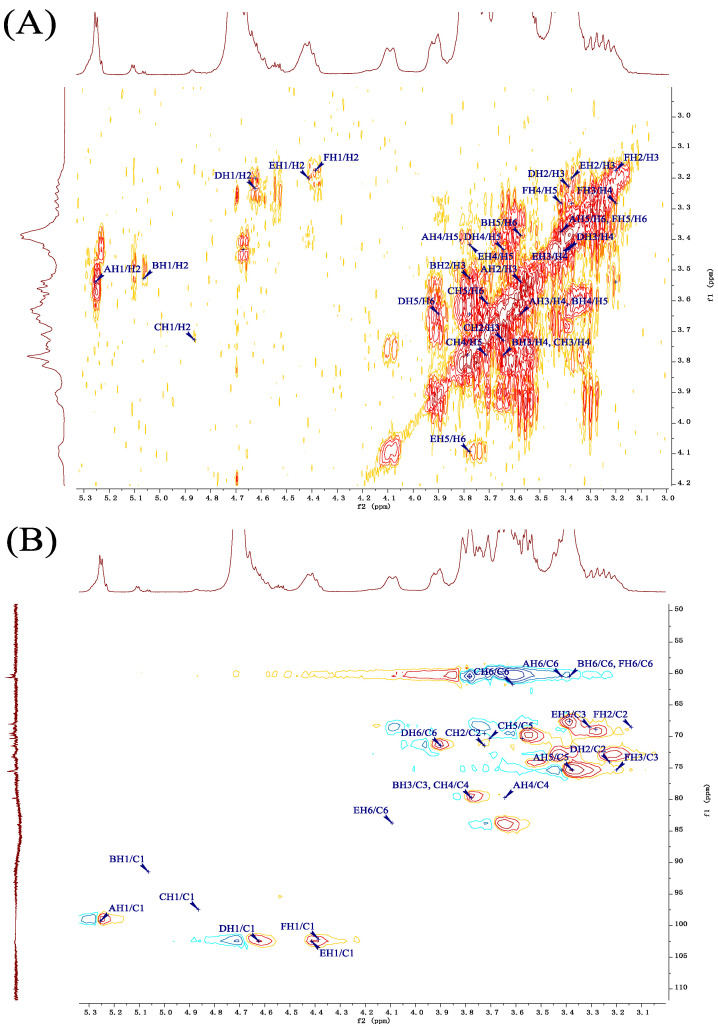

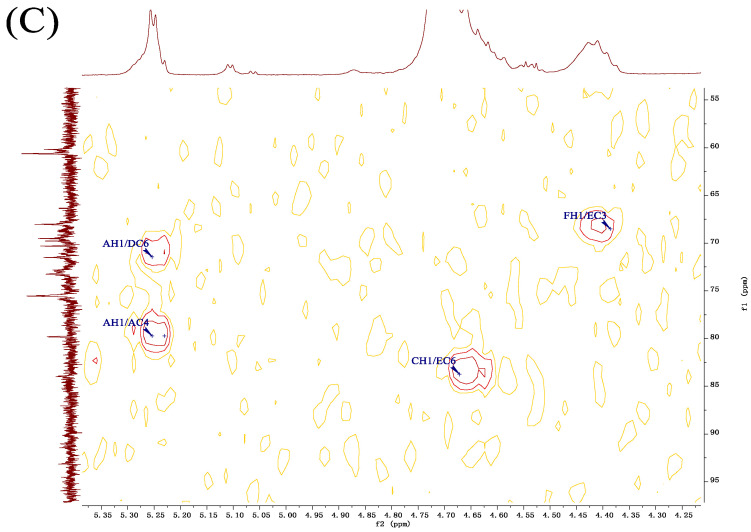
2D-NMR spectra of GLSP. (**A**) ^1^H–^1^H COSY spectrum; (**B**) HSQC spectrum; (**C**) HMBC spectrum.

The polysaccharide structure was analyzed using two-dimensional HSQC NMR spectroscopy (Figure 5B), identifying six major hydrogen–carbon correlation signals. These include anomeric proton signals at 5.25/99.26 ppm, 5.06/91.48 ppm, 4.86/97.48 ppm, 4.62/102.50 ppm, 4.41/102.50 ppm, and 4.39/102.16 ppm, corresponding to distinct sugar residues in the polysaccharide chain. Integrating the methylation analysis and the signal intensities from the HSQC spectra, the resonances were assigned to specific sugar residues as follows: 5.25/99.26 ppm corresponds to α–Glcp linked at position 4, 5.06/91.48 ppm to α–Glcp terminal residue, 4.86/97.48 ppm to β–Glcp linked at position 1, and so forth for other identified signals.

Using multiple 2D NMR spectra, we deduced the hydrogen–carbon (H-C) signal positions for six residual sugars in the polysaccharide. For instance, the 5.25 ppm peak was attributed to the anomeric proton (H-1) of the α-1,4-linked glucopyranose (Glcp) unit, as confirmed by ^1^H–^1^H COSY (Figure 5A) and HSQC spectra (Figure 5B), both showing a signal at 3.54 ppm for H-2. Further ^1^H–^1^H COSY and HSQC analyses established the carbon signals for this sugar unit up to C-6, with assignments at 3.54/69.50 ppm for C-2, 3.58/70.31 ppm for C-3, and continuing similarly for C-4 through C-6 with their respective hydrogen and carbon signals detailed in Table 3.

The HMBC technique (Figure 5C) is crucial for identifying hydrogen–carbon correlations across 2–3 bonds and elucidating glucosidic linkages in polysaccharides. For instance, in the β-1,3-linked glucopyranose (Glcp) residue, the HMBC spectrum revealed significant signals at 4.39/68.51 ppm, indicative of a linkage to C-3 in a β-1,3,6-Glcp unit [32]. Similarly, signals at 4.62/83.74 ppm helped confirm a glycosidic bond between the C-1 of a β-1,6-Glcp residue and C-6 of a β-1,3,6-Glcp unit. Furthermore, correlation signals at 5.25/71.44 and 5.25/79.72 ppm established linkages involving the anomeric carbon of an α-1,4-Glcp residue to the C-6 and C-4 of adjacent units, elucidating the complex branching pattern of the polysaccharide.

**Figure 6:**
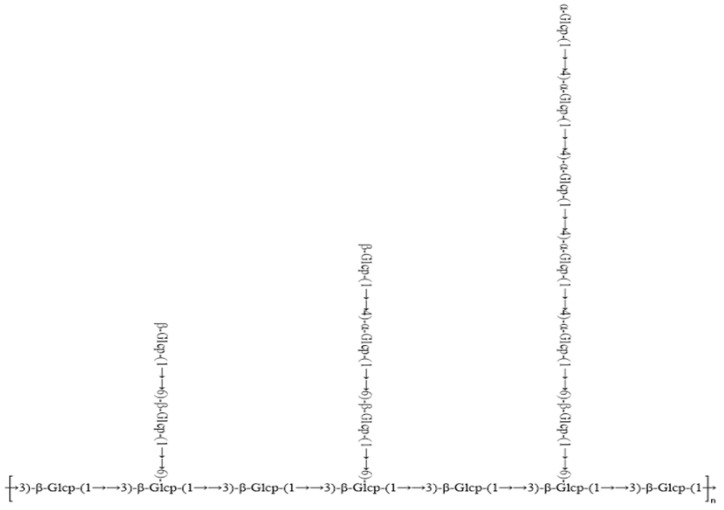
Structure of GLSP.

Based on the above analysis, according to the results of monosaccharide composition proportion and methylation of the glucan mainly by α–Glcp–(1→, →4) –α–Glcp–(1→, β–Glcp–(1→, →6)–β–Glcp–(1→, →3)–β–Glcp–(1→ and →3,6)–β–Glcp–(1→ six kinds of connection mode, its molar ratio is about 1:5:2:3:4:3; the binding weight average molecular weight Mw is 3291 Da. The main structure diagram of the polysaccharide is shown in Figure 6 (based on the above six residual sugar structures, *n* = 1 or 2).

### 3.4. Inhibitory Effect of GLSP on the Growth of Four Mouse Tumor Models

At the end of the experiment, four kinds of mouse tumor models were stripped, their tumor weight was calculated, and the tumor inhibition rate was calculated. The models of mouse sarcoma S180 (subcutaneous cell axillary inoculation), mouse Lewis lung cancer (subcutaneous toe inoculation), mouse liver cancer H22 (subcutaneous cell axillary inoculation), and mouse colon cancer C26 (subcutaneous cell axillary inoculation) were reviewed, as shown in Table 4, Table 5, Table 6 and Table 7, which revealed notable trends in tumor inhibition rates across varying doses of GLSP administration. Specifically, we observed a dose-dependent increase in tumor inhibition rate, with higher doses (50 mg/kg and 25 mg/kg) demonstrating particularly significant efficacy. Notably, the high and medium dose administration groups consistently achieved tumor inhibition rates exceeding 30%, meeting the criteria for immune antitumor drugs. Furthermore, our results exhibited reproducibility, bolstering the reliability of our findings.

Comparing our findings with the existing literature, we observe consistent evidence supporting the antitumor efficacy of *Ganoderma lucidum* spore polysaccharides (GLSPs) across a spectrum of tumor models. Previous studies have highlighted the GLSP’s ability to induce tumor cell apoptosis and impede tumor growth by modulating diverse molecular pathways. Moreover, in vivo investigations have demonstrated the GLSP’s capacity to effectively inhibit tumor progression, further reinforcing our own observations.

Our study contributes novel insights into the antitumor potential of the GLSP, particularly within the context of multiple tumor models, thereby emphasizing its versatility as a promising therapeutic agent. Additionally, the absence of drug-related toxicity observed in our experiments underscores the favorable safety profile of the GLSP, a critical aspect for its potential clinical translation.

## 4. Conclusions

Our study elucidated the preparation process and structural characteristics of *Ganoderma lucidum* spore polysaccharides (GLSPs), shedding light on their potential as a therapeutic agent against cancer. Through meticulous analysis, we identified the GLSP’s molecular weight, monosaccharide composition, and structural configurations, providing valuable insights into its composition and properties. Notably, the GLSP exhibited significant inhibitory effects on the growth of various mouse tumor models, including sarcoma S180, Lewis lung cancer, liver cancer H22, and colon cancer C26. These findings underscore the GLSP’s potential as a promising candidate for the development of antitumor pharmaceuticals or health products, offering prospects characterized by minimal adverse reactions and favorable therapeutic effects. In extending the current state of research, our study contributes to a deeper understanding of the GLSP’s antitumor efficacy and structural characteristics. Moving forward, further experiments could delve into elucidating the specific molecular mechanisms underlying the GLSP’s antitumor activity, potentially through in-depth molecular and cellular studies. Additionally, exploring the GLSP’s efficacy in combination with existing anti-cancer therapies or investigating its effects on other tumor types could provide valuable insights for future clinical applications. In conclusion, our study underscores the significant potential of the GLSP as a novel antitumor therapeutic agent, offering promising avenues for future research and clinical translation in the fight against cancer.

## Figures and Tables

**Table 1:** Monosaccharide compositions (%) of GLSPs.

Monosaccharide Compositions	Ratio (%)
Galactose	3.1
Glucose	87.5
Mannose	0.7
Fructose	8.7

**Table 2:** GC-MS analysis of methylated GLSPs.

RT (min)	Mass Fragments (m/z)	Type of Linkage
12.655	45, 59, 74, 87, 101, 117, 145, 161, 162, 182, 205, 220, 265, 269	Glcp–(1→
13.314	45, 58, 71, 87, 101, 102, 117, 129, 159, 161, 173, 201, 217, 233, 234, 277	→3)–Glcp–(1→ →4)–Glcp–(1→
13.529	43, 59, 71, 82, 87, 88, 99, 101, 117, 129, 159, 161, 173, 197, 226, 233, 247, 263, 266	→6)–Glcp–(1→
14.150	45, 58, 71, 85, 87, 101, 110, 117, 127, 136, 149, 159, 173, 192, 222, 233, 239, 248, 267, 292	→3,6)–Glcp–(1→

**Table 3:** Chemical shift assignment of glycosidic linkages in D_2_O.

Glycosidic Linkage		1	2	3	4	5	6
→4-α-Glcp-(1→ (A)	H C	5.25 99.26	3.54 69.50	3.58 70.31	3.64 79.72	3.38 75.32	3.42 60.46
α-t-Glcp-(1→ (B)	H C	5.06 91.48	3.53 74.03	3.78 79.72	3.64 75.32	3.58 70.31	3.39 60.46
β-t-Glcp-(1→ (C)	H C	4.86 97.48	3.73 71.44	3.64 74.03	3.78 79.72	3.71 70.31	3.61 61.74
→6)-β-Glcp-(1→ (D)	H C	4.62 102.50	3.23 74.03	3.39 67.56	3.43 72.74	3.64 75.32	3.90 71.44
→3,6)-β-Glcp-(1→ (E)	H C	4.41 102.50	3.20 72.74	3.38 68.51	3.42 72.74	3.78 74.68	4.09 83.74
→3)-β-Glcp-(1→ (F)	H C	4.39 102.16	3.17 68.51	3.20 75.32	3.28 68.85	3.42 72.74	3.38 60.46

**Table 4:** Antitumor effect of GLSPs for injection on mouse sarcoma S180 (cell axillary subcutaneous inoculation model).

Group	Mice (*n*)	Tumor (g, Mean ± SD)	TGI (%)
NS	10	4.07 ± 0.51	
CTX (100 mg/kg)	10	0.413 ± 0.20 **	89.85
GLSP (12.5 mg/kg)	10	3.11 ± 0.45 **	23.39
GLSP (25 mg/kg)	10	2.77 ± 0.55 **	31.94
GLSP (50 mg/kg)	10	2.67 ± 0.48 **	34.40

** *p* < 0.01, compared with the negative control group. The same as in the following table. *n*, number of mice.

**Table 5:** Antitumor effect of GLSPs for injection on mouse Lewis lung cancer (subcutaneous inoculation model of toe).

Group	Mice (*n*)	Tumor (g, Mean ± SD)	TGI (%)
NS	8	1.31 ± 0.23	
CTX (100 mg/kg)	8	0.108 ± 0.07 **	91.75
GLSP (12.5 mg/kg)	8	1.00 ± 0.14 **	23.66
GLSP (25 mg/kg)	8	0.891 ± 0.16 **	31.98
GLSP (50 mg/kg)	8	0.835 ± 0.09 **	36.25

**Table 6:** Antitumor effect of GLSPs for injection on mouse liver cancer H22 (cell axillary subcutaneous inoculation model).

Group	Mice (*n*)	Tumor (g, Mean ± SD)	TGI (%)
NS	10	3.61 ± 0.48	
5-Fu (30 mg/kg)	10	1.86 ± 0.36 **	48.48
GLSP (12.5 mg/kg)	10	2.66 ± 0.40 **	26.32
GLSP (25 mg/kg)	10	2.48 ± 0.35 **	31.30
GLSP (50 mg/kg)	10	2.39 ± 0.28 **	33.80

**Table 7:** Antitumor effect of GLSPs for injection on mouse colon cancer C26 (cell axillary subcutaneous inoculation model).

Group	Mice (*n*)	Tumor (g, Mean ± SD)	TGI (%)
NS	8	3.60 ± 0.50	
DDP (7 mg/kg)	8	0.30 ± 0.10 **	91.66
GLSP (12.5 mg/kg)	8	2.40 ± 0.20 **	36.11
GLSP (25 mg/kg)	8	2.30 ± 0.20 **	37.68
GLSP (50 mg/kg)	8	2.20 ± 0.20 **	38.88

## Data Availability

The original contributions presented in the study are included in the article, further inquiries can be directed to the corresponding author.
